# A dataset of human-inedible byproduct feeds consumed by dairy cows in the United States

**DOI:** 10.1016/j.dib.2021.107358

**Published:** 2021-09-08

**Authors:** Mary Beth de Ondarza, Juan M. Tricarico

**Affiliations:** a Paradox Nutrition, LLC, United States; b Dairy Management Inc., United States

**Keywords:** Feed byproducts, Dairy cows

## Abstract

Dairy cows convert human-indigestible forages and byproducts nutrients into edible food for humans [1]. Because of microbiota located in their rumen, dairy cows can digest fibrous forages and feeds which are not exploited by humans and monogastric animals like pigs. Dairy cows in the U.S. have been fed byproduct feeds as part of their diet for decades [2], [3]. Dairy nutritionists use complex nutrition models to develop economical and nutritious diets composed of grains, byproduct feeds, and forages. Accurate, complete, up-to-date information on byproduct feed consumption by dairy cows would be useful for both public and private researchers seeking to understand the type and extent of byproduct usage on US dairies. In collaboration with the American Feed Industry Association (AFIA), a survey was sent to US feed company representatives inquiring about the types and amounts of byproducts sold as dairy cow feed during the last year, the number of lactating cows serviced, the amount of milk produced by these lactating cows, and the states where these cows were located. A similar survey was sent to practicing US dairy nutritionists inquiring about their typical daily feeding rates of byproduct feeds by type, the number of cows consuming these byproducts, the amount of milk produced by the lactating cows, and the states where these cows were located. Survey data are representative of 33.5% of US lactating cows and 35.7% of US milk production in 2019 [4]. Amounts of each type of byproduct feed consumed per US milking cow (including replacement heifers and dry cows) and per kg of milk produced were calculated for the US and its four regions [5]. Total 2019 regional and US byproduct consumption by type was calculated. Nutrient compositions of each byproduct feed were reported.

## Specifications Table

**Table:** 

Subject	Animal Science
Specific subject area	Amounts of byproduct feeds by type consumed by dairy cows
Type of data	Table
How data were acquired	Survey (see Experimental Design, Materials and Methods)
Data format	analyzed
Parameters for data collection	US feed company representatives and practicing US dairy nutritionists were surveyed in 2019 regarding byproduct feed usage on dairy farms.
Description of data collection	US feed company representatives and practicing US dairy nutritionists were contacted directly via e-mail and submitted survey information online via the SurveyPlanet platform.
Data source location	United States of America
Data accessibility	With the article
Related research article	de Ondarza, M.B., Tricarico, J.M., 2021, Nutritional contributions and non-CO_2_ greenhouse gas emissions from human-inedible byproduct feeds consumed by dairy cows in the United States, J. Cleaner Prod. 315, 128125 https://doi.org/10.1016/j.jclepro.2021.128125

## Value of the Data

• Byproduct feed usage impacts farm profitability, forage production including land and water needs, and environmental sustainability of US dairies. Byproduct consumption by dairy cows also affects requirements for alternative waste disposal.
• Accurate, complete, and up-to-date information on byproduct feed consumption by US dairy cows would be useful for both public and private researchers and advisors including economists, agronomists, nutritionists, and environmentalists seeking to understand the type and extent of byproduct usage by US dairies.
• This survey data on byproduct feed consumption by US dairy cows could be used by environmental scientists who require inputs for life-cycle assessment models to evaluate the carbon footprint of US dairies, by economists working to assess and improve farm profitability, by milk processors seeking to understand sources of dairy cow nutrients, and by those responsible for valuating and disposing agricultural and agro-industrial byproducts.

## 1 Data Description

**Supplementary Table 1**. Feed byproducts included in surveys

**Supplementary Table 2.**

Rows 3 to 63: List of 61 byproduct feeds

Columns 2 to 6: Kg per Milking Cow per day (AF) for US and its regions

Columns 7 to 11: Metric tons fed (AF) based on 2019 Cow Numbers for US and its regions

**Supplementary Table 3.**

Rows 3 to 63: List of 61 byproduct feeds

Columns 2 to 6: Kg per Kg Milk (AF) for US and its regions

Columns 7 to 11: Metric tons fed (AF) based on 2019 Milk Production for US and its regions

**Supplementary Table 4.**

Rows 3 to 63: List of 61 byproduct feeds

Columns 2 to 13: Nutrient composition of byproduct feeds: DM (Dry Matter), Ash, CP (Crude Protein), NDF (Neutral Detergent Fiber), ADF (Acid Detergent Fiber), ADL (Acid Detergent Lignin), Starch, Sugar, EE (Ether Extract or Fat), TFA (Total Fatty Acids), GE (Gross Energy), ME (Metabolizable Energy).

## 2 Experimental Design, Materials and Methods

Survey questions were posed to US feed industry representatives and US dairy nutritionists via an online survey platform (SurveyPlanet). The survey sent to 407 US feed company representatives in July and October of 2019 included the following four questions:

1. List the states where your facility(s) delivers milking cow (lactating dairy cows and their associated replacement heifers and dry cows) feed.
2. From a list of byproducts (Supplementary Table 1), how many tons of each of these byproducts did your facility(s) purchase and sell as milking cow feed in the last year?
3. What is the average daily number of lactating cows (dry cows and replacement heifers excluded) that consumed feed from your facility(s) in the last year?
4. What is the average daily milk production (pounds/cow/d) of all of the lactating cows consuming feed from your facility(s) in the last year?

The survey sent to 336 practicing US dairy nutritionists in September 2019 included the following seven questions:

1. List the states where you are providing nutritional services for milking cows (lactating dairy cows and their associated replacement heifers and dry cows).
2. From the list of byproducts (Supplementary Table 1), estimate the percentage of milking cows that you feed that consume each one.
3. From the list of byproducts, what is the average pounds/cow/d of each byproduct in your lactating cow diets (dry cows and replacement heifers excluded)?
4. From the list of byproducts, what is the average pounds/cow/d of each byproduct in your dry cow diets?
5. From the list of byproducts, what is the average pounds/heifer/d of each byproduct in your growing heifer diets?
6. What is the average daily milk production (pounds/cow/d) of all of the lactating cows that you provide nutritional services for?
7. What is the average daily number of lactating cows that you provide nutritional services for?

Responses from both surveys were used to calculate kg of each byproduct fed per milking cow (Supplementary Table 2) and per kg of milk (Supplementary Table 3). A “milking cow” was defined as a lactating cow plus associated dry cows and replacement heifers. Using the responses provided by US feed industry representatives, kg of each byproduct fed per milking cow equaled the kg of each byproduct sold to dairy farms in 2019 (including replacement heifers and dry cows) divided by the number of lactating cows serviced. For example, a feed company that sold 4,535,929 kg (5000 tons) of beet pulp to service 50,000 lactating cows in 2019, would equate to 0.25 kg beet pulp/milking cow/day. Further, if average milk production was 30 kg/lactating cow/day, this would equate to 0.008 kg beet pulp per kg of milk produced. The information regarding daily byproduct intake of lactating cows, dry cows, and heifers was collected separately in the survey administered to US dairy nutritionists. The following assumptions were used to calculate kg of each byproduct fed per milking cow from the responses by US dairy nutritionists: a 60-day dry period, a herd cull rate of 37% [6], a 10% replacement heifer cull rate [7], and replacement heifer byproduct consumption from 4 to 24 months of age. Therefore, byproduct/milking cow (kg/cow/d) = (((BP_lact_ + BP_dry_ + BP_heifer_)/2.20462) × %C) where: BP_lact_ = byproduct/lactating cow (lbs/cow/d); BP_dry_ = byproduct/dry cow (lbs/cow/d) × 0.20; BP_heifer_ = byproduct/heifer (lbs/heifer/d) × (0.37 × 1.1 × (20/12)); %C = % of each nutritionist's cows consuming each byproduct. For example, if a dairy nutritionist fed 2 pounds/day of beet pulp to lactating cows, 2 pounds/day of beet pulp to dry cows, and 1 pound/day of beet pulp to heifers on all their dairy farms, this would equate to 1.4 kg of beet pulp/milking cow/day (((2 + (2 × 0.20) + (1 × 0.68))/2.20462) × 100%). Further, if average milk production was 30 kg/cow/day, this would equate to 0.047 kg beet pulp per kg of milk produced.

Survey information was categorized by region based on the states where each respondent shipped feed or provided nutritional services to estimate BP consumption on a regional basis (Northeast, Midwest, South, and West) [5]. Regional averages were weighed according to the average daily number of lactating cows serviced by each survey respondent. The 2019 USDA regional milk cow numbers and milk production [8] were used to calculate weighted averages of BP fed per milking cow and per kg of milk, respectively. The weighted regional and US averages were multiplied by 2019 USDA milk cow numbers and milk production [8], respectively, to estimate metric tons of BP fed based on cow numbers and milk production.

## Ethics Statement

All survey participants were informed in their letter of invitation to participate in the survey that: “The information will be held completely confidential and only provided as a total of all the data.” Confidentiality for the dairy nutritionist and feed company is important since their specific diet formulations are their source of competitive advantage. The SurveyPlanet platform grants Survey Creators “a license to disclose and share the survey questions, responses, and results (“Survey Data”) with their friends, colleagues, and other third parties”. This study was conducted in compliance with the National Dairy Council ‘Guiding Principles for Research and Communication of Results’ [9].

## CRediT Author Statement

**Mary Beth de Ondarza:** Investigation, Data Curation, Formal Analysis, Methodology, Validation, Writing – original Draft; **Juan M. Tricarico:** Conceptualization, Funding acquisition, Methodology, Project administration, Supervision, Validation, Writing – review & editing.

## Declaration of Competing Interest

The authors declare that they have no known competing financial interests or personal relationships which have or could be perceived to have influenced the work reported in this article.

## Appendix Supplementary materials

Image, application 1

Image, application 2

Image, application 3

Image, application 4
