# Supplementary material for: A dataset of human-inedible byproduct feeds consumed by dairy cows in the United States
Source: Data Brief. 2021 Sep 8;38:107358. doi: 10.1016/j.dib.2021.107358 (PMC8446783; doi:10.1016/j.dib.2021.107358)
Supplement: Supplementary file 1 [file mmc1.docx]

Supplementary Table 1. Feed byproducts included in AFIA and dairy nutritionist surveys

| AFIA Survey | | |
| --- | --- | --- |
| Almond Hulls (Prunus dulcis) | Cottonseed (Whole) (Gossypium) | Rice Bran (Oryza sativa) |
| Bakery Waste (Triticum) | Cottonseed Hulls (Gossypium) | Rice Hulls (Oryza sativa) |
| Beet Pulp (Beta vulgaris) | Cottonseed Meal (Gossypium) | Rice Mill Feed (Oryza sativa) |
| Blood Meal (Sanguis) | Fat - Animal | Safflower Meal (Carthamus tinctorius) |
| Brewer’s Grains, Dry (Hordeum vulgare) | Fat - Vegetable | Soybean Flour (Glycine max) |
| Brewer’s Cond. Solubles (Hordeum vulgare) | Feather Meal | Soybean Hulls (Glycine max) |
| Candy | Fish Meal | Soybean Meal (Glycine max) |
| Canola Meal (Brassica napus) | Hominy Feed (Zea mays) | Soybean Meal – Treated (Glycine max) |
| Canola Meal – Treated (Brassica napus) | Linseed Meal (Linum usitatissimum) | Sugar (Sucrose) |
| Cereal | Meat Meal | Sunflower Meal (Helianthus) |
| Chocolate (Cacao) | Meat and Bone Meal | Wheat Bran (Triticum) |
| Citrus Pulp - Dry | Molasses – Beet (Beta vulgaris) | Wheat Distillers Grains – Dry (Triticum) |
| Corn Distillers – Dry (Zea mays) | Molasses – Cane (Saccharum officinarum) | Wheat Flour (Triticum) |
| Corn Germ Meal (Zea mays) | Oat Hulls (Avena sativa) | Wheat Midds (Triticum) |
| Corn Gluten Feed – Dry (Zea mays) | Oat Mill Feed (Avena sativa) | Wheat Millrun (Triticum) |
| Corn Gluten Meal (Zea mays) | Peanut Hulls (Arachis hypogaea) | Wheat Red Dog Flour (Triticum) |
| Corn Starch (Zea mays) | Peanut Meal (Arachis hypogaea) | Wheat Shorts (Triticum) |
|  |  | Whey, Dry |
|  |  |  |
| Dairy Nutritionist Survey | | |
| Almond Hulls(Prunus dulcis) | Corn Starch (Zea mays) | Potato Waste (Solanum tuberosum) |
| Bakery Waste (Triticum) | Corn Steep Liquor (Zea mays) | Rice Bran (Oryza sativa) |
| Beet Pulp (Beta vulgaris) | Cottonseed (Whole) (Gossypium) | Rice Hulls (Oryza sativa) |
| Blood Meal (Sanguis) | Cottonseed Hulls (Gossypium) | Rice Mill Feed (Oryza sativa) |
| Brewer’s Grains, Dry (Hordeum vulgare) | Cottonseed Meal (Gossypium) | Safflower Meal (Carthamus tinctorius) |
| Brewer’s Grains, Wet (Hordeum vulgare) | Fat - Animal | Soybean Hulls (Glycine max) |
| Candy | Fat - Vegetable | Soybean Meal (Glycine max) |
| Canola Meal (Brassica napus) | Feather Meal | Soybean Meal – Treated (Glycine max) |
| Canola Meal – Treated (Brassica napus) | Fish Meal | Sugar (Sucrose) |
| Cereal | Hominy Feed (Zea mays) | Sunflower Meal (Helianthus) |
| Chocolate (Cacao) | Linseed Meal (Linum usitatissimum) | Wheat Bran (Triticum) |
| Citrus Pulp – Dry | Malt Sprouts (Hordeum vulgare) | Wheat Distillers Grains – Dry (Triticum) |
| Citrus Pulp - Wet | Meat Meal | Wheat Distillers Grains – Wet (Triticum) |
| Corn Cannery Waste – Wet (Zea mays) | Meat and Bone Meal | Wheat Flour (Triticum) |
| Corn Distillers – Dry (Zea mays) | Molasses – Beet (Beta vulgaris) | Wheat Midds (Triticum) |
| Corn Distillers – Wet (Zea mays) | Molasses – Cane (Saccharum officinarum) | Wheat Millrun (Triticum) |
| Corn Germ Meal (Zea mays) | Oat Hulls (Avena sativa) | Wheat Red Dog Flour (Triticum) |
| Corn Gluten Feed – Dry (Zea mays) | Oat Mill Feed (Avena sativa) | Wheat Shorts (Triticum) |
| Corn Gluten Feed – Wet (Zea mays) | Peanut Hulls (Arachis hypogaea) | Whey, Dry |
| Corn Gluten Meal (Zea mays) | Peanut Meal (Arachis hypogaea) | Whey, Liquid |
|  |  | Whey, Condensed |
