# Supplementary material for: A dataset of human-inedible byproduct feeds consumed by dairy cows in the United States
Source: Data Brief. 2021 Sep 8;38:107358. doi: 10.1016/j.dib.2021.107358 (PMC8446783; doi:10.1016/j.dib.2021.107358)
Supplement: Supplementary file 2 [file mmc2.docx]

Supplementary Table 2. Byproduct feeds consumed daily per milking cow and annually by US milking cows in 2019

|  | Kg Per Milking Cow per Day (AF) | | | | | Metric Tons Fed (AF) Based on 2019 Cow Numbers^1^ | | | | |
| --- | --- | --- | --- | --- | --- | --- | --- | --- | --- | --- |
|  | US^2^ | Midwest | Northeast | South | West | US^2^ | Midwest | Northeast | South | West |
| Almond Hulls | 0.551 | 0.043 | 0.000 | 0.000 | 1.385 | 1878076 | 51616 | 0 | 0 | 1826459 |
| Bakery Waste | 0.096 | 0.068 | 0.280 | 0.010 | 0.081 | 327487 | 82408 | 134707 | 4115 | 106257 |
| Beet Pulp | 0.083 | 0.048 | 0.228 | 0.007 | 0.086 | 284201 | 58491 | 109494 | 2769 | 113447 |
| Blood Meal | 0.142 | 0.116 | 0.130 | 0.330 | 0.115 | 485630 | 140314 | 62397 | 131829 | 151090 |
| Brewer's Grain Dry | 0.021 | 0.000 | 0.044 | 0.000 | 0.038 | 71588 | 0 | 21319 | 0 | 50269 |
| Brewer's Grain Wet | 0.448 | 0.241 | 0.370 | 0.927 | 0.520 | 1525586 | 291511 | 177837 | 370761 | 685477 |
| Candy | 0.005 | 0.000 | 0.021 | 0.000 | 0.006 | 18479 | 0 | 9925 | 0 | 8554 |
| Canola Meal | 1.510 | 0.865 | 0.983 | 0.223 | 2.684 | 5146420 | 1045089 | 472962 | 89363 | 3539006 |
| Canola Meal (Trt) | 0.032 | 0.002 | 0.186 | 0.000 | 0.013 | 109093 | 2085 | 89479 | 0 | 17530 |
| Cereal | 0.326 | 0.857 | 0.000 | 0.000 | 0.056 | 1109988 | 1035828 | 29 | 0 | 74131 |
| Chocolate | 0.003 | 0.000 | 0.000 | 0.000 | 0.006 | 8681 | 0 | 128 | 0 | 8554 |
| Citrus Pulp Dry | 0.081 | 0.092 | 0.072 | 0.225 | 0.030 | 274459 | 110720 | 34696 | 90038 | 39005 |
| Citrus Pulp Wet | 0.392 | 0.000 | 0.000 | 0.000 | 1.013 | 1335820 | 0 | 0 | 0 | 1335820 |
| Corn Cannery Waste | 0.566 | 1.365 | 0.007 | 0.000 | 0.209 | 1928400 | 1649520 | 3213 | 0 | 275667 |
| Corn Distillers' Dry | 1.214 | 0.650 | 0.652 | 1.594 | 1.821 | 4138312 | 784744 | 313601 | 637921 | 2402046 |
| Corn Distillers' Wet | 1.007 | 0.084 | 0.043 | 1.424 | 2.078 | 3431294 | 101126 | 20707 | 569542 | 2739918 |
| Corn Germ Meal | 0.059 | 0.000 | 0.000 | 0.000 | 0.151 | 199458 | 0 | 0 | 0 | 199458 |
| Corn Gluten Feed Dry | 0.529 | 0.206 | 0.518 | 1.892 | 0.415 | 1802769 | 249207 | 249165 | 756927 | 547469 |
| Corn Gluten Feed Wet | 0.092 | 0.180 | 0.021 | 0.000 | 0.065 | 312628 | 217227 | 10200 | 0 | 85200 |
| Corn Gluten Meal 60% | 0.019 | 0.013 | 0.081 | 0.000 | 0.007 | 63869 | 15976 | 38994 | 0 | 8899 |
| Corn Starch | 0.022 | 0.004 | 0.134 | 0.000 | 0.003 | 74338 | 5416 | 64645 | 0 | 4277 |
| Corn Steep Liquor | 0.047 | 0.009 | 0.004 | 0.000 | 0.112 | 160120 | 10551 | 2054 | 0 | 147514 |
| Cottonseed Whole | 0.792 | 0.342 | 0.177 | 1.378 | 1.251 | 2699871 | 413082 | 85175 | 551395 | 1650218 |
| Cottonseed Hulls | 0.019 | 0.031 | 0.002 | 0.010 | 0.018 | 65194 | 37058 | 899 | 3805 | 23432 |
| Cottonseed Meal | 0.051 | 0.000 | 0.008 | 0.239 | 0.057 | 174513 | 194 | 3672 | 95482 | 75165 |
| Fat - Animal | 0.024 | 0.029 | 0.011 | 0.001 | 0.032 | 82800 | 34919 | 5424 | 558 | 41899 |
| Fat - Vegetable | 0.006 | 0.002 | 0.034 | 0.000 | 0.002 | 21304 | 2630 | 16181 | 153 | 2341 |
| Feather Meal | 0.005 | 0.001 | 0.008 | 0.000 | 0.011 | 18485 | 609 | 3668 | 0 | 14208 |
| Fish Meal | 0.001 | 0.001 | 0.001 | 0.000 | 0.001 | 3139 | 1587 | 478 | 0 | 1074 |
| Hominy Feed | 0.072 | 0.062 | 0.007 | 0.152 | 0.080 | 244237 | 74837 | 3417 | 60863 | 105120 |
| Linseed Meal | 0.048 | 0.001 | 0.001 | 0.000 | 0.123 | 163750 | 909 | 638 | 23 | 162180 |
| Malt Sprouts | 0.052 | 0.055 | 0.015 | 0.000 | 0.077 | 175709 | 66778 | 7140 | 0 | 101790 |
| Meat Meal | 0.000 | 0.000 | 0.000 | 0.000 | 0.000 | 214 | 0 | 0 | 0 | 214 |
| Meat and Bone Meal | 0.013 | 0.035 | 0.000 | 0.000 | 0.000 | 42815 | 42442 | 159 | 0 | 214 |
| Molasses - Beet | 0.056 | 0.095 | 0.006 | 0.000 | 0.056 | 190615 | 114301 | 2678 | 0 | 73636 |
| Molasses - Cane | 0.152 | 0.159 | 0.254 | 0.120 | 0.118 | 517589 | 192313 | 121976 | 48115 | 155184 |
| Oat Hulls | 0.033 | 0.081 | 0.024 | 0.000 | 0.003 | 113716 | 98106 | 11655 | 0 | 3954 |
| Oat Mill Feed | 0.001 | 0.001 | 0.000 | 0.000 | 0.002 | 3774 | 609 | 0 | 0 | 3165 |
| Peanut Hulls | 0.005 | 0.003 | 0.000 | 0.001 | 0.010 | 17739 | 3855 | 0 | 382 | 13501 |
| Peanut Meal | 0.004 | 0.000 | 0.000 | 0.000 | 0.009 | 12317 | 0 | 0 | 0 | 12317 |
| Potato Waste | 0.036 | 0.026 | 0.000 | 0.000 | 0.070 | 124053 | 31766 | 0 | 0 | 92287 |
| Rice Bran | 0.024 | 0.000 | 0.000 | 0.000 | 0.062 | 81651 | 0 | 0 | 0 | 81651 |
| Rice Hulls | 0.004 | 0.005 | 0.000 | 0.000 | 0.005 | 12603 | 5951 | 0 | 0 | 6651 |
| Rice Mill Feed | 0.008 | 0.000 | 0.000 | 0.000 | 0.022 | 28484 | 0 | 0 | 0 | 28484 |
| Safflower Meal | 0.007 | 0.000 | 0.000 | 0.000 | 0.018 | 24307 | 0 | 0 | 0 | 24307 |
| Soybean Hulls | 0.264 | 0.123 | 0.472 | 0.385 | 0.281 | 899968 | 149113 | 226902 | 153978 | 369975 |
| Soybean Meal | 1.020 | 0.760 | 1.530 | 2.281 | 0.691 | 3477523 | 917684 | 736094 | 912457 | 911288 |
| Soybean Meal (Trt) | 0.345 | 0.306 | 0.673 | 0.306 | 0.273 | 1176179 | 369995 | 323669 | 122517 | 359997 |
| Sugar | 0.005 | 0.000 | 0.028 | 0.001 | 0.002 | 16486 | 604 | 13395 | 349 | 2138 |
| Sunflower Meal | 0.010 | 0.000 | 0.000 | 0.000 | 0.026 | 33758 | 84 | 0 | 0 | 33674 |
| Wheat Bran | 0.031 | 0.000 | 0.008 | 0.000 | 0.077 | 105720 | 7 | 3890 | 0 | 101823 |
| Wheat Distillers - Dry | 0.029 | 0.000 | 0.000 | 0.000 | 0.075 | 98797 | 0 | 0 | 0 | 98797 |
| Wheat Distillers - Wet | 0.069 | 0.000 | 0.000 | 0.000 | 0.179 | 236598 | 0 | 0 | 0 | 236598 |
| Wheat Flour | 0.006 | 0.000 | 0.000 | 0.000 | 0.016 | 20529 | 0 | 0 | 0 | 20529 |
| Wheat Midds | 0.252 | 0.356 | 0.524 | 0.066 | 0.116 | 860441 | 429661 | 251999 | 26358 | 152422 |
| Wheat Mill Run | 0.080 | 0.010 | 0.000 | 0.000 | 0.197 | 271940 | 11566 | 0 | 0 | 260374 |
| Wheat Red Dog | 0.020 | 0.000 | 0.113 | 0.000 | 0.010 | 67872 | 76 | 54452 | 0 | 13344 |
| Wheat Shorts | 0.001 | 0.000 | 0.000 | 0.000 | 0.003 | 4277 | 0 | 0 | 0 | 4277 |
| Whey Dry | 0.002 | 0.003 | 0.000 | 0.000 | 0.002 | 6449 | 3650 | 64 | 15 | 2720 |
| Whey Acid | 0.340 | 0.140 | 0.693 | 0.000 | 0.497 | 1157200 | 168807 | 333348 | 0 | 655045 |
| Whey Condensed | 0.832 | 0.054 | 0.453 | 3.140 | 0.984 | 2836305 | 64817 | 217650 | 1256344 | 1297494 |

^1^ Calculated based on 2019 USDA cow numbers [8]

^2^ US average amounts of each BP consumed were calculated using regional averages weighted according to 2019 USDA regional milk cow numbers
