# Supplementary material for: A dataset of human-inedible byproduct feeds consumed by dairy cows in the United States
Source: Data Brief. 2021 Sep 8;38:107358. doi: 10.1016/j.dib.2021.107358 (PMC8446783; doi:10.1016/j.dib.2021.107358)
Supplement: Supplementary file 3 [file mmc3.docx]

Supplementary Table 3. Byproduct feeds consumed per kg of milk and in annual metric tons according to US milk production in 2019

|  | Kg Per Kg Milk (AF) | | | | | Metric Tons Fed (AF) Based on 2019 Milk Production^1^ | | | | |
| --- | --- | --- | --- | --- | --- | --- | --- | --- | --- | --- |
|  | US^2^ | Midwest | Northeast | South | West | US^2^ | Midwest | Northeast | South | West |
| Almond Hulls | 0.015 | 0.001 | 0.000 | 0.000 | 0.038 | 1517965 | 38204 | 0 | 0 | 1479761 |
| Bakery Waste | 0.003 | 0.002 | 0.008 | 0.000 | 0.002 | 253921 | 61696 | 104353 | 3309 | 84562 |
| Beet Pulp | 0.002 | 0.001 | 0.006 | 0.000 | 0.002 | 228373 | 48796 | 86320 | 2227 | 91030 |
| Blood Meal | 0.004 | 0.003 | 0.004 | 0.009 | 0.003 | 366795 | 105339 | 47457 | 94125 | 119875 |
| Brewer's Grain Dry | 0.001 | 0.000 | 0.001 | 0.000 | 0.001 | 59484 | 0 | 15737 | 0 | 43746 |
| Brewer's Grain Wet | 0.012 | 0.006 | 0.010 | 0.026 | 0.014 | 1186014 | 223053 | 133640 | 280099 | 549222 |
| Candy | 0.000 | 0.000 | 0.001 | 0.000 | 0.000 | 14480 | 0 | 7700 | 0 | 6780 |
| Canola Meal | 0.041 | 0.022 | 0.027 | 0.006 | 0.072 | 4044217 | 783495 | 363328 | 65110 | 2832283 |
| Canola Meal (Trt) | 0.001 | 0.000 | 0.005 | 0.000 | 0.000 | 85604 | 1838 | 69879 | 0 | 13888 |
| Cereal | 0.008 | 0.021 | 0.000 | 0.000 | 0.001 | 802447 | 743489 | 23 | 0 | 58935 |
| Chocolate | 0.000 | 0.000 | 0.000 | 0.000 | 0.000 | 6878 | 0 | 98 | 0 | 6780 |
| Citrus Pulp Dry | 0.002 | 0.003 | 0.002 | 0.006 | 0.001 | 214472 | 89259 | 26273 | 68021 | 30918 |
| Citrus Pulp Wet | 0.011 | 0.000 | 0.000 | 0.000 | 0.028 | 1083315 | 0 | 0 | 0 | 1083315 |
| Corn Cannery Waste | 0.014 | 0.033 | 0.000 | 0.000 | 0.006 | 1406533 | 1181221 | 2629 | 0 | 222682 |
| Corn Distillers' Dry | 0.033 | 0.017 | 0.018 | 0.042 | 0.049 | 3234089 | 618402 | 245393 | 457651 | 1912643 |
| Corn Distillers' Wet | 0.027 | 0.002 | 0.001 | 0.037 | 0.056 | 2692146 | 77517 | 15733 | 404963 | 2193933 |
| Corn Germ Meal | 0.002 | 0.000 | 0.000 | 0.000 | 0.004 | 158679 | 0 | 0 | 0 | 158679 |
| Corn Gluten Feed Dry | 0.014 | 0.005 | 0.015 | 0.049 | 0.011 | 1367233 | 190259 | 196118 | 540050 | 440806 |
| Corn Gluten Feed Wet | 0.002 | 0.005 | 0.001 | 0.000 | 0.002 | 235777 | 160861 | 7365 | 0 | 67551 |
| Corn Gluten Meal 60% | 0.000 | 0.000 | 0.002 | 0.000 | 0.000 | 49362 | 11896 | 30384 | 0 | 7083 |
| Corn Starch | 0.001 | 0.000 | 0.004 | 0.000 | 0.000 | 57189 | 4225 | 49573 | 0 | 3390 |
| Corn Steep Liquor | 0.001 | 0.000 | 0.000 | 0.000 | 0.003 | 126616 | 8101 | 1531 | 0 | 116984 |
| Cottonseed Whole | 0.021 | 0.009 | 0.005 | 0.036 | 0.034 | 2091459 | 307438 | 64612 | 394851 | 1324558 |
| Cottonseed Hulls | 0.001 | 0.001 | 0.000 | 0.000 | 0.000 | 52380 | 29880 | 663 | 3019 | 18817 |
| Cottonseed Meal | 0.001 | 0.000 | 0.000 | 0.006 | 0.002 | 131045 | 179 | 2651 | 67891 | 60324 |
| Fat - Animal | 0.001 | 0.001 | 0.000 | 0.000 | 0.001 | 65626 | 27701 | 4004 | 422 | 33500 |
| Fat - Vegetable | 0.000 | 0.000 | 0.001 | 0.000 | 0.000 | 17375 | 2070 | 13038 | 123 | 2144 |
| Feather Meal | 0.000 | 0.000 | 0.000 | 0.000 | 0.000 | 14460 | 477 | 2721 | 0 | 11262 |
| Fish Meal | 0.000 | 0.000 | 0.000 | 0.000 | 0.000 | 2477 | 1271 | 345 | 0 | 861 |
| Hominy Feed | 0.002 | 0.002 | 0.000 | 0.004 | 0.002 | 190233 | 58628 | 2467 | 45980 | 83157 |
| Linseed Meal | 0.001 | 0.000 | 0.000 | 0.000 | 0.003 | 130160 | 1125 | 460 | 18 | 128556 |
| Malt Sprouts | 0.001 | 0.001 | 0.000 | 0.000 | 0.002 | 136883 | 50853 | 5344 | 0 | 80686 |
| Meat Meal | 0.000 | 0.000 | 0.000 | 0.000 | 0.000 | 170 | 0 | 0 | 0 | 170 |
| Meat and Bone Meal | 0.000 | 0.001 | 0.000 | 0.000 | 0.000 | 31806 | 31521 | 115 | 0 | 170 |
| Molasses - Beet | 0.001 | 0.002 | 0.000 | 0.000 | 0.001 | 143304 | 83137 | 1933 | 0 | 58233 |
| Molasses - Cane | 0.004 | 0.004 | 0.007 | 0.003 | 0.003 | 404265 | 145464 | 94185 | 36527 | 128088 |
| Oat Hulls | 0.001 | 0.002 | 0.001 | 0.000 | 0.000 | 83780 | 72030 | 8612 | 0 | 3139 |
| Oat Mill Feed | 0.000 | 0.000 | 0.000 | 0.000 | 0.000 | 2935 | 427 | 0 | 0 | 2509 |
| Peanut Hulls | 0.000 | 0.000 | 0.000 | 0.000 | 0.000 | 15261 | 3108 | 0 | 308 | 11846 |
| Peanut Meal | 0.000 | 0.000 | 0.000 | 0.000 | 0.000 | 9764 | 0 | 0 | 0 | 9764 |
| Potato Waste | 0.001 | 0.001 | 0.000 | 0.000 | 0.002 | 96589 | 22766 | 0 | 0 | 73823 |
| Rice Bran | 0.001 | 0.000 | 0.000 | 0.000 | 0.002 | 65103 | 0 | 0 | 0 | 65103 |
| Rice Hulls | 0.000 | 0.000 | 0.000 | 0.000 | 0.000 | 10062 | 4788 | 0 | 0 | 5274 |
| Rice Mill Feed | 0.000 | 0.000 | 0.000 | 0.000 | 0.001 | 22579 | 0 | 0 | 0 | 22579 |
| Safflower Meal | 0.000 | 0.000 | 0.000 | 0.000 | 0.000 | 19325 | 0 | 0 | 0 | 19325 |
| Soybean Hulls | 0.007 | 0.003 | 0.013 | 0.011 | 0.007 | 702192 | 116042 | 174991 | 116849 | 294309 |
| Soybean Meal | 0.027 | 0.020 | 0.042 | 0.060 | 0.018 | 2665823 | 708139 | 567187 | 663025 | 727473 |
| Soybean Meal (Trt) | 0.009 | 0.008 | 0.019 | 0.009 | 0.007 | 920818 | 286719 | 253253 | 94133 | 286713 |
| Sugar | 0.000 | 0.000 | 0.001 | 0.000 | 0.000 | 12688 | 486 | 10244 | 264 | 1695 |
| Sunflower Meal | 0.000 | 0.000 | 0.000 | 0.000 | 0.001 | 27259 | 64 | 0 | 0 | 27196 |
| Wheat Bran | 0.001 | 0.000 | 0.000 | 0.000 | 0.002 | 85819 | 5 | 2839 | 0 | 82974 |
| Wheat Distillers - Dry | 0.001 | 0.000 | 0.000 | 0.000 | 0.002 | 78423 | 0 | 0 | 0 | 78423 |
| Wheat Distillers - Wet | 0.002 | 0.000 | 0.000 | 0.000 | 0.005 | 187545 | 0 | 0 | 0 | 187545 |
| Wheat Flour | 0.000 | 0.000 | 0.000 | 0.000 | 0.000 | 16273 | 0 | 0 | 0 | 16273 |
| Wheat Midds | 0.007 | 0.009 | 0.015 | 0.002 | 0.003 | 659337 | 318969 | 196899 | 21197 | 122273 |
| Wheat Mill Run | 0.002 | 0.000 | 0.000 | 0.000 | 0.005 | 225677 | 9324 | 0 | 0 | 216353 |
| Wheat Red Dog | 0.001 | 0.000 | 0.003 | 0.000 | 0.000 | 50915 | 58 | 40280 | 0 | 10577 |
| Wheat Shorts | 0.000 | 0.000 | 0.000 | 0.000 | 0.000 | 3390 | 0 | 0 | 0 | 3390 |
| Whey Dry | 0.000 | 0.000 | 0.000 | 0.000 | 0.000 | 5149 | 2934 | 46 | 12 | 2156 |
| Whey Acid | 0.009 | 0.004 | 0.019 | 0.000 | 0.013 | 898124 | 125736 | 254291 | 0 | 518098 |
| Whey Condensed | 0.022 | 0.001 | 0.013 | 0.081 | 0.026 | 2147404 | 46486 | 167745 | 893300 | 1039873 |

^1^ Calculated based on 2019 USDA milk production [8]

^2^ US average amounts of each BP consumed were calculated using regional averages weighted according to 2019 USDA regional milk cow numbers and milk production.
