# Supplementary material for: A dataset of human-inedible byproduct feeds consumed by dairy cows in the United States
Source: Data Brief. 2021 Sep 8;38:107358. doi: 10.1016/j.dib.2021.107358 (PMC8446783; doi:10.1016/j.dib.2021.107358)
Supplement: Supplementary file 4 [file mmc4.docx]

Supplementary Table 4. Nutrient composition of feed byproducts (%DM)^1^ (CNCPS 6.55, NDS Ver. 3.9.7.11, Van Amburgh et al., 2015)

|  | DM | Ash | CP | NDF | ADF | ADL | Starch | Sugar | EE | TFA | GE,Mcal/kg | ME,Mcal/kg |
| --- | --- | --- | --- | --- | --- | --- | --- | --- | --- | --- | --- | --- |
| Almond Hulls | 87.0 | 7.4 | 6.0 | 33.0 | 28.8 | 11.2 | 3.2 | 34.5 | 3.0 | 1.3 | 4.13 | 1.92 |
| Bakery Waste | 94.8 | 4.1 | 13.0 | 10.5 | 4.6 | 1.5 | 46.3 | 13.4 | 9.1 | 8.1 | 4.68 | 3.27 |
| Beet Pulp | 91.0 | 12.2 | 9.8 | 41.6 | 25.9 | 4.4 | 3.3 | 10.0 | 1.0 | 0.8 | 3.88 | 2.03 |
| Blood Meal | 90.0 | 2.4 | 95.0 | 0.0 | 0.0 | 0.0 | 0.0 | 1.1 | 1.5 | 1.3 | 5.51 | 3.78 |
| Brewer's Grain Dry | 92.7 | 4.2 | 24.0 | 44.7 | 25.7 | 5.8 | 12.4 | 2.8 | 9.6 | 8.3 | 4.86 | 2.70 |
| Brewer's Grain Wet | 24.5 | 4.3 | 29.0 | 47.1 | 23.0 | 6.3 | 4.4 | 2.6 | 9.5 | 7.6 | 4.92 | 2.67 |
| Candy | 89.3 | 5.3 | 10.6 | 16.2 | 7.6 | 2.7 | 14.7 | 41.5 | 8.7 | 8.5 | 4.58 | 3.11 |
| Canola Meal | 88.0 | 7.6 | 41.7 | 28.8 | 18.4 | 7.7 | 5.8 | 8.1 | 3.3 | 2.6 | 4.64 | 2.60 |
| Canola Meal (Trt) | 95.0 | 6.7 | 37.8 | 27.8 | 19.0 | 6.5 | 5.8 | 8.0 | 10.7 | 8.5 | 5.00 | 2.92 |
| Cereal | 93.3 | 3.6 | 11.8 | 15.5 | 5.5 | 2.1 | 49.0 | 14.5 | 2.3 | 2.3 | 4.33 | 2.90 |
| Chocolate | 89.3 | 2.7 | 11.7 | 17.2 | 9.1 | 2.2 | 12.9 | 35.3 | 16.2 | 10.3 | 5.09 | 3.14 |
| Citrus Pulp Dry | 88.6 | 6.9 | 7.3 | 23.9 | 17.5 | 2.6 | 4.3 | 24.8 | 2.9 | 1.7 | 4.16 | 2.52 |
| Citrus Pulp Wet | 19.0 | 6.9 | 7.3 | 23.9 | 17.5 | 2.6 | 4.3 | 24.8 | 2.9 | 1.7 | 4.16 | 2.52 |
| Corn Cannery Waste | 22.0 | 4.4 | 9.5 | 64.6 | 34.9 | 3.0 | 2.2 | 1.1 | 6.8 | 5.1 | 4.50 | 2.34 |
| Corn Distillers' Dry | 88.8 | 5.9 | 30.3 | 33.6 | 16.0 | 4.5 | 5.5 | 4.3 | 14.5 | 11.6 | 5.13 | 3.07 |
| Corn Distillers' Wet | 32.3 | 5.3 | 32.0 | 32.0 | 16.3 | 4.9 | 3.7 | 4.9 | 12.0 | 9.6 | 5.05 | 3.01 |
| Corn Germ Meal | 93.3 | 3.8 | 24.8 | 39.3 | 14.1 | 2.2 | 18.6 | 1.4 | 12.0 | 8.0 | 5.01 | 2.98 |
| Corn Gluten Feed Dry | 89.7 | 7.5 | 25.3 | 34.7 | 10.8 | 1.9 | 14.2 | 4.1 | 4.2 | 3.4 | 4.46 | 2.77 |
| Corn Gluten Feed Wet | 41.1 | 6.8 | 22.5 | 36.7 | 11.7 | 2.2 | 14.7 | 4.1 | 4.3 | 2.2 | 4.45 | 2.61 |
| Corn Gluten Meal 60% | 91.6 | 3.5 | 65.5 | 7.1 | 5.3 | 1.9 | 16.2 | 2.5 | 2.7 | 2.5 | 5.11 | 3.75 |
| Corn Starch | 92.0 | 0.1 | 0.0 | 0.0 | 0.0 | 0.0 | 97.8 | 2.0 | 0.1 | 0.1 | 4.20 | 3.69 |
| Corn Steep Liquor | 44.8 | 15.3 | 42.6 | 0.5 | 0.2 | 0.2 | 15.7 | 7.4 | 1.7 | 0.0 | 4.24 | 3.02 |
| Cottonseed Whole | 92.0 | 4.2 | 23.5 | 50.3 | 40.1 | 12.9 | 0.4 | 2.7 | 18.6 | 18.3 | 5.32 | 2.90 |
| Cottonseed Hulls | 92.0 | 2.8 | 6.1 | 84.7 | 65.0 | 22.6 | 1.2 | 1.5 | 3.5 | 3.1 | 4.35 | 1.00 |
| Cottonseed Meal | 92.0 | 8.2 | 42.3 | 30.6 | 20.2 | 7.4 | 1.2 | 6.0 | 3.1 | 3.1 | 4.61 | 2.71 |
| Fat - Animal | 99.0 | 0.0 | 0.0 | 0.0 | 0.0 | 0.0 | 0.0 | 0.0 | 100.0 | 88.0 | 9.40 | 7.07 |
| Fat - Vegetable | 99.0 | 0.0 | 0.0 | 0.0 | 0.0 | 0.0 | 0.0 | 0.0 | 100.0 | 88.0 | 9.40 | 7.07 |
| Feather Meal | 93.3 | 3.0 | 85.0 | 2.0 | 1.0 | 0.0 | 0.0 | 0.0 | 10.0 | 7.8 | 5.78 | 3.96 |
| Fish Meal | 90.0 | 19.0 | 63.3 | 8.3 | 1.6 | 0.3 | 0.0 | 0.0 | 9.4 | 6.4 | 4.78 | 3.34 |
| Hominy Feed | 88.4 | 3.0 | 11.0 | 19.0 | 6.0 | 1.2 | 54.3 | 2.7 | 4.9 | 4.5 | 4.48 | 3.04 |
| Linseed Meal | 88.0 | 6.5 | 33.0 | 31.4 | 18.3 | 7.5 | 8.9 | 9.1 | 1.5 | 1.3 | 4.47 | 2.57 |
| Malt Sprouts | 93.0 | 6.9 | 24.6 | 43.8 | 18.0 | 2.9 | 7.5 | 10.2 | 1.9 | 1.5 | 4.35 | 2.53 |
| Meat Meal | 93.9 | 21.5 | 58.2 | 8.0 | 5.0 | 0.0 | 0.0 | 0.0 | 12.3 | 7.5 | 4.75 | 3.00 |
| Meat and Bone Meal | 95.4 | 26.0 | 54.5 | 7.2 | 4.0 | 0.0 | 0.0 | 0.0 | 12.3 | 7.4 | 4.51 | 2.82 |
| Molasses - Beet | 75.0 | 11.0 | 8.5 | 0.0 | 0.0 | 0.0 | 0.0 | 70.0 | 1.0 | 0.6 | 3.91 | 2.81 |
| Molasses - Cane | 73.0 | 11.0 | 5.8 | 0.0 | 0.0 | 0.0 | 0.0 | 70.0 | 1.0 | 0.6 | 3.87 | 2.77 |
| Oat Hulls | 93.5 | 5.2 | 5.9 | 71.4 | 39.3 | 6.0 | 11.7 | 1.8 | 2.3 | 1.8 | 4.18 | 1.86 |
| Oat Mill Feed | 90.0 | 6.0 | 8.0 | 40.0 | 20.0 | 2.0 | 34.3 | 6.9 | 1.2 | 1.0 | 4.12 | 2.44 |
| Peanut Hulls | 93.1 | 6.0 | 11.4 | 62.5 | 51.4 | 20.6 | 3.9 | 10.4 | 4.4 | 4.3 | 4.34 | 1.42 |
| Peanut Meal | 92.0 | 6.7 | 48.9 | 14.0 | 6.3 | 1.4 | 11.0 | 13.4 | 1.6 | 1.6 | 4.69 | 3.33 |
| Potato Waste | 93.6 | 4.0 | 10.7 | 12.1 | 8.3 | 3.1 | 61.6 | 2.1 | 5.9 | 4.6 | 4.49 | 2.96 |
| Rice Bran | 91.0 | 11.9 | 14.0 | 30.1 | 17.3 | 6.5 | 24.9 | 4.7 | 14.1 | 12.0 | 4.63 | 2.67 |
| Rice Hulls | 93.5 | 5.2 | 5.9 | 71.4 | 39.3 | 6.0 | 11.7 | 1.8 | 2.3 | 1.8 | 4.18 | 1.86 |
| Rice Mill Feed | 89.8 | 10.5 | 12.0 | 25.4 | 15.0 | 4.4 | 33.9 | 3.7 | 14.0 | 11.9 | 4.65 | 2.84 |
| Safflower Meal | 93.6 | 5.3 | 26.6 | 50.9 | 37.8 | 14.0 | 1.3 | 4.2 | 4.3 | 3.9 | 4.57 | 2.08 |
| Soybean Hulls | 91.0 | 5.0 | 12.1 | 65.9 | 47.5 | 3.0 | 1.7 | 2.2 | 3.0 | 1.6 | 4.31 | 2.16 |
| Soybean Meal | 90.0 | 6.7 | 51.5 | 10.0 | 6.8 | 0.8 | 1.9 | 10.9 | 2.8 | 2.7 | 4.79 | 3.50 |
| Soybean Meal (Trt) | 90.1 | 7.4 | 52.0 | 24.0 | 5.2 | 0.7 | 0.7 | 11.0 | 1.7 | 1.7 | 4.71 | 3.29 |
| Sugar | 98.0 | 0.0 | 0.0 | 0.0 | 0.0 | 0.0 | 0.0 | 100.0 | 0.0 | 0.0 | 4.20 | 3.70 |
| Sunflower Meal | 93.0 | 8.0 | 40.2 | 38.0 | 31.0 | 8.9 | 1.8 | 5.9 | 2.0 | 1.0 | 4.53 | 2.38 |
| Wheat Bran | 88.8 | 5.8 | 17.0 | 44.0 | 15.0 | 3.5 | 21.8 | 4.7 | 4.5 | 4.0 | 4.43 | 2.56 |
| Wheat Distillers - Dry | 92.4 | 5.3 | 39.3 | 32.0 | 15.1 | 4.3 | 3.2 | 4.3 | 14.5 | 11.5 | 5.28 | 3.21 |
| Wheat Distillers - Wet | 32.3 | 5.3 | 39.3 | 32.0 | 15.1 | 4.3 | 3.2 | 4.3 | 14.5 | 11.5 | 5.28 | 3.21 |
| Wheat Flour | 95.0 | 3.0 | 10.0 | 6.0 | 2.0 | 0.1 | 77.0 | 1.6 | 1.3 | 1.2 | 4.28 | 3.11 |
| Wheat Midds | 89.0 | 6.2 | 18.4 | 38.0 | 13.3 | 3.2 | 22.3 | 5.1 | 4.5 | 3.9 | 4.43 | 2.64 |
| Wheat Mill Run | 95.0 | 2.0 | 15.0 | 25.0 | 8.0 | 1.3 | 33.2 | 2.8 | 3.5 | 3.5 | 4.51 | 3.04 |
| Wheat Red Dog | 88.6 | 3.7 | 20.1 | 27.0 | 7.8 | 1.4 | 37.3 | 5.6 | 4.1 | 3.6 | 4.54 | 3.00 |
| Wheat Shorts | 89.0 | 2.0 | 15.0 | 25.0 | 5.0 | 1.3 | 35.9 | 2.8 | 3.5 | 3.5 | 4.51 | 3.04 |
| Whey Dry | 95.0 | 10.0 | 14.6 | 0.0 | 0.0 | 0.0 | 0.0 | 70.0 | 0.7 | 0.1 | 4.02 | 2.69 |
| Whey Acid | 7.0 | 10.5 | 14.2 | 0.0 | 0.0 | 0.0 | 0.0 | 70.0 | 0.7 | 0.1 | 3.99 | 2.87 |
| Whey Condensed | 20.0 | 10.0 | 14.6 | 0.0 | 0.0 | 0.0 | 0.0 | 70.0 | 0.7 | 0.1 | 4.02 | 2.89 |

^1^ DM (Dry Matter), Ash, CP (Crude Protein), NDF (Neutral Detergent Fiber), ADF (Acid Detergent Fiber), ADL (Acid Detergent Lignin), Starch, Sugar, EE (Ether Extract or Fat), TFA (Total Fatty Acids), GE (Gross Energy), ME (Metabolizable Energy)
